# Toxoplasma Gondii Replication During Belatacept Treatment in Kidney Transplantation: A Case Report and a Review of the Literature

**DOI:** 10.3390/genes16040391

**Published:** 2025-03-29

**Authors:** Raffaella Vigilante, Raafiah Izhar, Rossella Di Paola, Ananya De, Rosa Maria Pollastro, Giovanna Capolongo, Giulio Viceconte, Mariadelina Simeoni

**Affiliations:** 1 Department of Translation Medical Sciences, University of Campania “Luigi Vanvitelli”, 80131 Naples, Italy; raffaellavigilanteav@gmail.com (R.V.); rosamaria.pollastro@unicampania.it (R.M.P.); giovanna.capolongo@unicampania.it (G.C.); 2 Department of Mental and Physical Health and Preventive Medicine, University of Campania “Luigi Vanvitelli”, 80138 Naples, Italy; rosella.dipaola@unicampania.it (R.D.P.); ananyade150111997@gmail.com (A.D.); 3 Department of Infectious Diseases, University Hospital ‘Federico II’, 80131 Naples, Italy; giulio.viceconte@unina.it

**Keywords:** belatacept, kidney transplant, neurotoxoplasmosis, guanylate binding proteins, immunosuppressive therapy

## Abstract

Belatacept is a chimeric protein that acts as a selective blocker of T-lymphocyte co-stimulation. It has been proposed for the prevention of kidney transplant rejection. This paper reports a literature review on pharmacological characteristics of belatacept and genetic factors influencing its efficacy and safety profile. A severe case of neurotoxoplasmosis observed in a kidney transplant recipient (KTR) treated with belatacept is also described. It appears that the interference of belatacept on guanylate binding proteins (GBPs) expression in antigen-presenting cells (APC) cytoplasm could be involved in Toxoplasma gondii (Toxo-g) reactivation in seropositive KTRs. Additionally, genetic variations in immune regulatory genes encoding CTLA-4 and Blimp-1 may influence individual susceptibility to infection and immune modulation under belatacept therapy. In conclusion, we highlight the importance of drug avoidance and/or increased surveillance in Toxo-g IgG-positive KTR. We also retain that further studies on the host defense pathways involved in the surveillance of opportunistic pathogens in KTR are strongly desirable.

## 1. Introduction

Kidney transplant is recognized to be the renal replacement therapy associated with better survival and quality of life [1]. However, immunosuppressive agents used to prevent graft rejection also have the reversal effect of increasing both cancer [2,3] and infectious risk [4]. Belatacept is a chimeric protein acting as a selective blocker of T cell co-stimulation that has been recently proposed as a non-nephrotoxic maintenance immunosuppressant in kidney transplant recipients (KTR). The use of belatacept, a T-cell-targeting immunosuppressant, comes with the risk of increased infection and malignancy. However, only few data are available on its safety profile and mostly report a certain incidence of several cancer types and post-transplant lymphoproliferative disorders (PTLD) [5]. Belatacept, with its distinct mechanism of selectively blocking T-cell co-stimulation, offers an alternative to calcineurin inhibitors (CNIs) following kidney transplantation and may provide more advantages [6]. The BENEFIT (Belatacept Evaluation of Nephroprotection and Efficacy as First-line Immunosuppression Trial) and BENEFIT EXT (extended criteria donors) studies are the only randomized clinical trials that tested belatacept versus calcineurin inhibitors in a large population of KTRs. The BENEFIT study demonstrated that when used as an initial therapy post-transplant, belatacept enhanced long-term graft function, graft survival, and patient survival [6]. A randomized study by Budde et al. also reported benefits for graft function and a lower incidence of new donor-specific antibodies (1% versus 7%), switching stable KTRs from cyclosporine to belatacept [7].

Within opportunistic infections, only a few cases of tuberculosis and no cases of Toxoplasma gondii (Toxo-g) infection were reported in the belatacept arm [8]. Moreover, in a recent multicentric epidemiological study evaluating the rate of opportunistic infections after the switch to belatacept in KTR, no cases of Toxo-g infection were observed [9]. In our literature review on belatacept, we describe the drug’s mechanism of action, pharmacokinetics, pharmacodynamics, genetic factors, and safety profile and report a case of neurotoxoplasmosis in a KTR. In order to address this topic, we searched the available literature in all main libraries, such as PubMed, Cochrane, Medline, etc. We used the following keywords: ‘Belatacept and side effects’, ‘Belatacept and Toxoplasma gondii’, ‘Belatacept and opportunistic infection’, ‘Belatacept and Neurotoxoplasmosis’, ‘kidney transplant and Toxoplasmosis’, & Belatacept and immunosuppression. By selecting only English language articles, mainly original articles, reviews, and clinical trials, we conducted our narrative review for understanding the possible immunosuppressive pathways involved in belatacept-related neuroToxoplasmosis.

## 2. Belatacept Pharmacodynamics and Genetic Factors

Belatacept is an immunosuppressive drug currently approved for the treatment of KTR to prevent graft rejection, an event that is mediated by both CD4+ and CD8+ T cells and mostly occurs within the first post-transplantation year [10]. CNIs (like cyclosporine and tacrolimus) work by inhibiting calcineurin, leading to a broad suppression of the immune system. Belatacept, on the other hand, selectively blocks T-cell costimulation, preventing the full activation of T-cells. Studies have demonstrated that belatacept provides immunosuppressive efficacy while potentially avoiding long-term toxicities associated with CNIs [11].

**Figure 1:**
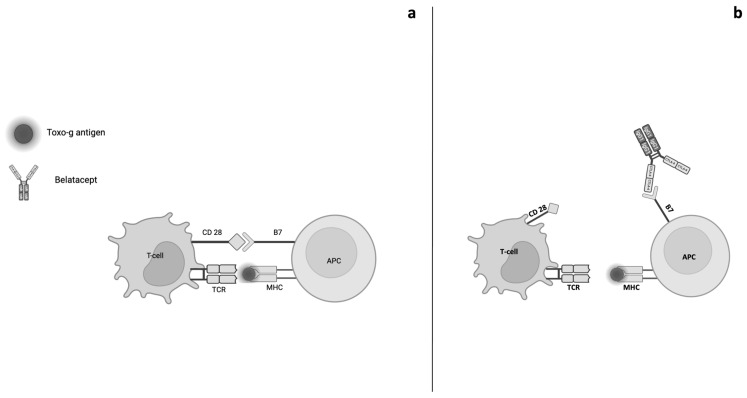
Comparison of T-cell co-activation and the mechanism of action of belatacept. (**a**). Activation of T-cell proliferation is determined by the occurrence of two events: 1. Interaction between the T-cell receptor (TCR) and antigen-binding major histocompatibility complex (MHC) expressed by the antigen-presenting cells (APCs); 2. Interaction between the CD28 protein, expressed by the T-cells, and the B7 ligands (CD80-CD86) present on the APCs. The concomitance of the two events allows the triggering of signals that promote T-cell proliferation. (**b**). The CTLA-4 component of belatacept binds to B7 with high affinity and thus effectively blocks signal 2.

Belatacept is a fusion protein composed of the Fc fragment of human IgG1 bound to the extracellular domain of cytotoxic T lymphocyte-associated antigen 4 (CTLA-4) and acts as a competitive antagonist or selective T lymphocyte blocker [10,12]. T lymphocytes play a central role in cell-mediated immunity and require two signals for full activation. The first signal is determined by T cell receptor (TCR) binding to antigen bound to the major histocompatibility complex (MHC) expressed on antigen-presenting cells (APC). The second signal is characterized by the binding of CD28 protein expressed on T lymphocytes with B7 (CD80 and CD86) ligands present on APC [10,13]. Belatacept mimics the action of the homologue of CD28, CTLA-4 [14]. In conclusion, belatacept acts by blocking the T cell co-stimulation and consequently the activation of T lymphocytes [15] (Figure 1).

The interplay between genetic factors and the immune response to belatacept underscores the necessity for personalized approaches in immunosuppressive therapy. Understanding these genetic influences can lead to a better stratification of patients based on their risk of rejection or infection, ultimately improving transplant outcomes and patient management strategies. Belatacept has been shown to significantly influence B cell responses, particularly through its effects on the transcription factor Blimp-1 (B lymphocyte-induced maturation protein 1). Research indicates that belatacept directly downregulates the expression of Blimp-1 in B cells, thereby impairing their ability to differentiate into antibody-secreting plasma cells. This inhibition leads to a reduced humoral immune response, which is particularly relevant in the context of renal transplantation, where a balanced antibody production is crucial for graft acceptance and protection against infections [16,17,18].

In addition to its role in B cells, Blimp-1 also influences T cell differentiation and function. For instance, it has been shown to repress the expression of IFN-γ in T cells, thereby modulating their effector functions [19]. This dual role of Blimp-1 in both B and T cells highlights its importance in the overall immune response, particularly in the context of infections. Toxo-g is an intracellular parasite that can lead to various immune responses, particularly affecting T cell functionality. Hwang et al. demonstrated that Blimp-1 mediates CD4 T cell exhaustion, which subsequently leads to the dysfunction of CD8 T cells during chronic Toxo-g infection [20].

The impact of belatacept on Treg (regulatory T cell) populations is another area of interest. While Tregs are essential for maintaining immune tolerance, studies have indicated that belatacept treatment may lead to a decrease in Treg function, as evidenced by reduced expression of FOXP3 and CD25, which are crucial for Treg activity [21]. This finding raises questions about the long-term implications of belatacept on immune regulation and graft acceptance, especially in sensitized patients who may have a more complex immune landscape [22]. Furthermore, the role of the gene encoding the CTLA-4 protein that belatacept mimics is also noteworthy. Genetic variants in the CTLA-4 locus have been implicated in various autoimmune disorders, suggesting that individual genetic backgrounds may affect the immune response to belatacept [23]. The translational implications of genetic variants in the CTLA-4 locus, particularly concerning their association with autoimmune diseases, are significant for personalized medicine and therapeutic interventions. Research by Khalaf et al. [24] has elucidated the connection between CTLA-4 polymorphisms and systemic lupus erythematosus (SLE) susceptibility, reinforcing the necessity of considering genetic backgrounds when evaluating risks for developing autoimmune diseases. The CTLA-4 +49A/G polymorphism, frequently studied in relation to SLE, demonstrates how specific genetic variations can alter immune responses, influencing disease progression and treatment responses. Another study found evidence supporting an association between CTLA-4 haplotypes and an increased risk of developing SLE, indicating that the expression and regulation of CTLA-4 could significantly affect disease progression and autoimmunity [25]. Moreover, research has shown abnormal CTLA-4 functionality in T-cells from SLE patients, indicating that even when CTLA-4 expression is present, its regulatory effects may be compromised, leading to ineffective control of T-cell activation and proliferation [26]. Furthermore, studies have highlighted that certain polymorphisms can lead to alterations in soluble CTLA-4 levels, which are crucial modulators of immune tolerance [27,28]. Specifically, the CT60 A/G polymorphism impacts the expression levels of soluble CTLA-4, potentially enhancing susceptibility to autoimmune diseases by altering the ratio of transmembrane to soluble forms of the protein [29]. Moreover, studies have demonstrated that CTLA-4 knockout mice exhibit uncontrolled T-cell proliferation, reinforcing the understanding of how genetic backgrounds can affect immune responses [30]. These genetic variants have significant implications in the context of belatacept, a fusion protein designed to inhibit the CD28-mediated costimulatory signal in T-cells. While belatacept has shown efficacy in preventing organ rejection, its effectiveness can vary among individuals with different genetic backgrounds, particularly in those with CTLA-4 deficiencies [31]. Overall, belatacept represents a significant advancement in immunosuppressive therapy for kidney transplantation, with its unique mechanism of action influencing gene expression and immune cell dynamics.

## 3. Belatacept Pharmacokinetics

The pharmacokinetics of belatacept were evaluated after multiple doses of 5 mg/kg or 10 mg/kg, and the highest tested concentrations were 136.3 μg/mL and 238.3 μg/mL, respectively. However, in KTR the recommended dose is 5 mg/kg/month without exceeding the total dose of 125 μg/mL/month. It was seen that the belatacept AUC after the single 5 mg/kg dose is 10,200 μg/h/mL. After the multiple doses of 5 mg/kg and 10 mg/Kg, the AUC instead was 13,500 μg h/mL and 21,200 μg h/mL, respectively [10,32]. The pharmacokinetics of belatacept are characterized by limited extravascular distribution and slow elimination. The reported drug half-life is 32–124 h in animals and 235 h in humans [32]. A cumulative 5 mL/h/kg dose in KTRs has about an 8–10 day half-life [8]. Population pharmacokinetics studies conducted on KTR revealed a correlation between belatacept clearance and high body weight. Conversely, age, sex, race, estimated GFR, albumin-related liver function, diabetes, and concomitant dialysis did not influence belatacept body clearance [33].

The Latek group reported that significantly lower levels of free CD86 receptors were present in the blood of belatacept-treated KTRs compared with both pre-transplant levels and the healthy controls. CD86 receptor saturation was related to belatacept dose/frequency, which remained above 80%. These results suggest that belatacept-mediated immunosuppression is primarily related to CD86 saturation compared with CD80 [34].

## 4. Adverse Events Related to Belatacept Treatment

Belatacept was approved in 2011 by the FDA for use in kidney transplantation as a non-nephrotoxic maintenance immunosuppressant. Its approval followed the publication of two pivotal studies, BENEFIT and BENEFIT-EXT. Both are presented as open-label, randomized, multicenter phase III studies that compared the efficacy and safety of more intensive (MI) or less intensive (LI) belatacept regimens versus cyclosporine A [35]. Anemia, urinary tract infection, hypertension, constipation, diarrhea, nausea, and peripheral edema were the most common adverse events (>25%) reported in both studies. In short-term (12 months) data, the belatacept group had a larger number of patients with malignancies (5%) compared to the cyclosporine group. Malignancies included Kaposi’s sarcoma, breast cancer, colon cancer, myelodysplastic syndrome, and prostate cancer. Also, long-term data showed that less than 3% of belatacept-treated patients developed post-transplant lymphoproliferative disorders (PTLD) with central nervous system involvement; this increased PTLD risk was primarily observed in patients with EBV-negative or CMV-positive status and those who received T-cell-depleting therapy. No patients treated with cyclosporine developed PTLD [8]. The overall incidence of bacterial, cytomegalovirus (CMV), BK polyomavirus, and fungal infections was similar among the in-study groups. A single case of progressive multifocal leukoencephalopathy (PML) was reported at one-year follow-up of the MI belatacept regimen [36]. A similar belatacept safety profile was confirmed in other reports thereafter [37,38,39].

Early use of belatacept revealed an increased risk of cerebral lymphoma, a severe complication with limited treatment options. However, this risk was mitigated by subsequently avoiding belatacept in patients with prior Epstein–Barr virus infection [40]. Immunosuppression introduced by belatacept can lead to insufficient control of EBV, especially in patients who lack pre-existing immunity to the virus [41,42,43,44]. Belatacept interferes with CD28-mediated T-cell activation, which is crucial for the immune response against latent viruses such as EBV. In EBV-naive patients, the use of belatacept can undermine the T-cell support necessary for controlling EBV-infected B cells, leading to an increased risk of post-transplant lymphoproliferative disease (PTLD) as unregulated EBV-infected lymphocytes proliferate without proper immune control [41,42,45].

## 5. Opportunistic Infections and Belatacept

Exploring the adverse side events reported in the literature, only a few cases of tuberculosis and no cases of Toxo-g infection had been reported within opportunistic infections [8]. In the course of Toxo-g infection in immunocompetent hosts, parasite antigen presentation by APC to T cells triggers the activation of IFNɣ and TNF1 [46] pathways able to induce Toxo-g latency. In immunosuppressed KTR treated with belatacept, these cascades are blocked, and we hypothesize that a severe Toxo-g reactivation or new infection may occur [9] (Figure 1).

Newly and supportively, we observed a case of severe neurotoxoplasmosis because of belatacept administration in a 71-year-old male kidney transplant recipient. In this regard, we read with great interest the paper by Fisch D. et al. [47] reporting a possible role of guanylate binding proteins (GBPs) in the surveillance of Toxo-g replication, as evaluated in a series of in vitro experiments, and found further support in this new insight from basic science.

**Figure 2:**
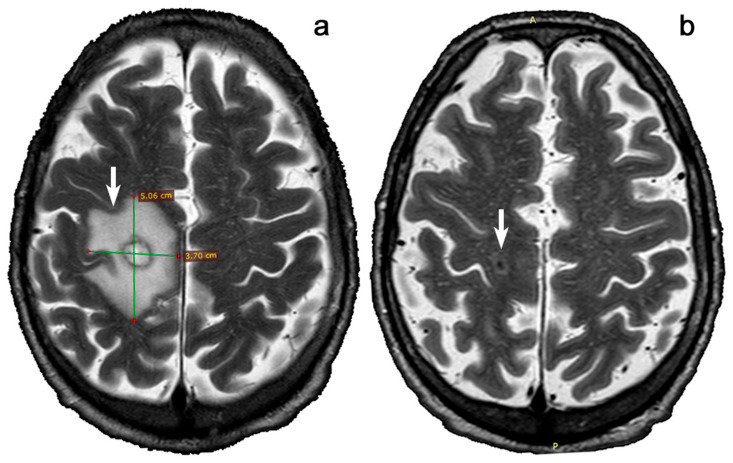
Brain MRI scan showing neuro-toxoplasmosis before and after treatment. (**a**). At baseline, a 5.3 × 3.7 cm nodular ring-enhancing lesion is evident in the right hemisphere (arrow); (**b**). The Toxo-g abscess, present at baseline in the right hemisphere, appears significantly reduced after a 6 weeks treatment course with trimethoprim/sulfamethoxazole (arrow).

Our patient was first-line immunosuppressed with tacrolimus and prednisone but showed a relevant rise in serum creatinine up to 3.8 mg/dl related to graft rejection at 7-month follow-up; so, he was treated with high-dose boluses of corticosteroids to manage a cell-mediated rejection. Furthermore, due to gastrointestinal adverse reactions to mycophenolate, the patient could not continue with a tacrolimus-based triple therapy regimen. For this reason, tacrolimus was suspended, a cycle of treatment with belatacept was started, and an improvement of renal function (serum creatinine decreased to 2.8 mg/dL) was soon after observed. After 8 months of therapy, due to the appearance of left brachial and crural hyposthenia, a brain CT and a brain MRI were consecutively performed. Imaging revealed a large nodular ring-enhancing formation in the right hemisphere, compatible with a differential diagnosis of brain abscess or neurolymphoma (Figure 2a).

A rachicentesis for liquor analysis was performed [48], and trimethoprim/sulfamethoxazole was started empirically [49]. A Toxo-g positivity was found in the liquor, and neuro-lymphoma was then excluded. Of note, pre-transplant Toxo-g IgG serostatus was positive for both the graft recipient and donor. Belatacept was promptly suspended, and trimethoprim/sulfamethoxazole was continued for 6 weeks. During the treatment, the patient showed progressive resolution of neurological symptoms with renal function stabilization. Post-treatment brain MRI showed significant regression of the Toxo-g-related abscess (Figure 2b).

**Figure 3:**
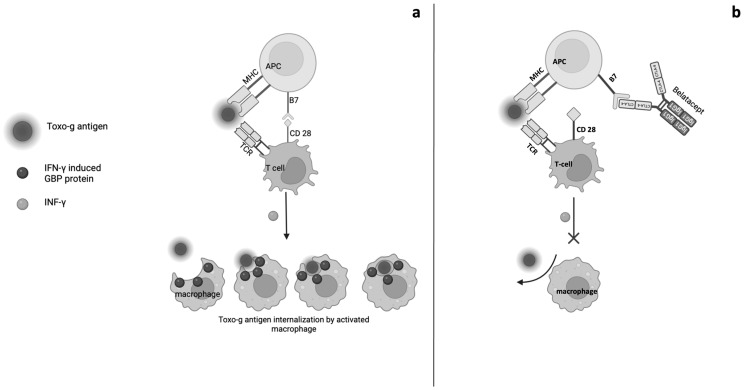
(**a**). Immune surveillance without belatacept: parasite recognition by dendritic cells (DCs) leads to CD4+ and CD8+ T cell costimulation with IFNγ production; IFNγ induces macrophage activation and GBPs expression on their Toxo-g parassitophorous vacuoles with Toxo-g lysis. (**b**). Interference of belatacept on cellular sources of IFNγ during Toxo-g infection: belatacept acts as a costimulation blocker of CD4+ and CD8+ T cells, resulting in a reduced IFNγ production with interference on GBP-mediated parasite lysis in macrophages.

The appearance in our patient of a severe form of neurotoxoplasmosis soon after belatacept initiation could indicate the direct pathogenetic role of the drug and could help in translating Fisch et al.’s [47] findings from an in vitro setting to humans. We retain that the mechanism of Toxo-g reactivation in our patient might have involved GBPs too. In fact, IFNɣ and TNF1 inhibition by belatacept might have impaired macrophage activation and polarization in response to Toxo-g, with the loss of GBPs inhibition on parasite growth inducing the described severe clinical evolution (Figure 3).

GBPs, such as GBP1, GBP2, GBP5, and others, play significant roles in the modulation of both innate and adaptive immunity. The interplay between GBPs and adaptive immunity is evident in their roles in modulating antigen-specific T-cell responses. GBPs can enhance the presentation of antigens derived from intracellular pathogens, thus improving T-cell activation and proliferation [50]. These proteins are well-known for their interferon-inducible expression and their ability to sense pathogens, thereby activating various immune effector mechanisms [50,51,52]. Several GBPs are recruited onto the *Toxoplasma* parasitophorous vacuole, and most are also required for restricting Toxo-g replication [53]. GBPs play a significant role in innate immunity. These proteins are inducible by IFN-γ and have been shown to assemble at the vacuoles where Toxo-g resides, promoting the destruction of the parasitophorous vacuole (PV) membrane. Research indicates that GBP2 and GBP7 specifically are crucial for disrupting the PV membrane, thereby facilitating the immune response against this intracellular pathogen [54,55].

Moreover, the interplay between Toxo-g and the immune system is complex, as the parasite can manipulate host immune responses. Toxo-g can induce changes in the migratory behavior of dendritic cells, which are pivotal in antigen presentation and T cell activation [56]. This manipulation may further exacerbate the exhaustion of T cells, as the altered immune environment can lead to persistent antigen exposure. By inhibiting T cell activation, belatacept may inadvertently affect the dynamics of T cell exhaustion and functionality, particularly in the context of Blimp-1 expression. Consequently, the role of Blimp-1 in this context is critical, as it not only influences T cell differentiation but also shapes the overall immune landscape during T. gondii infection [20]. The implications of Toxo-g actively infecting T-cells are multifaceted and can profoundly impact immune responses and disease mechanisms. Research highlights how Toxo-g can manipulate host immune responses by inducing the production of inflammatory cytokines, particularly interleukin-12 (IL-12), which plays a crucial role in T-cell differentiation [57]. Moreover, Toxo-g infection may contribute to the onset of immunological conditions such as type-1 diabetes. Autoimmunity against pancreatic β cells could be aggravated by the immune system’s aberrant activation in response to Toxo-g [58].

## 6. Conclusions

Belatacept is a relatively new immunosuppressant approved for preventing graft rejection in KTRs. In addition to its renal safety, this drug is associated with an increased oncologic and infectious risk. However, neurotoxoplasmosis has never been described in association with belatacept treatment. Translating from new knowledge to clinical practice on KTRs, even based on our case report, we conclude that further studies are needed to finely investigate the effects of immunosuppression and genetic factors on host defense pathways involved in the surveillance of opportunistic pathogens. The interference of belatacept on GBPs, Blimp-1, and CTLA-4 should also be investigated to understand individual susceptibility to infections. Understanding these genetic interactions could lead to personalized immunosuppressive strategies and improved patient outcomes. Closer surveillance and/or drug avoidance in Toxo-g seropositive KTRs should be considered in clinical practice.

## Data Availability

Not applicable.
